# Low-dose radiotherapy for painful osteoarthritis of the elderly: A multicenter analysis of 970 patients with 1185 treated sites

**DOI:** 10.1007/s00066-021-01816-y

**Published:** 2021-08-03

**Authors:** Alexander Rühle, Elisabeth Tkotsch, Rainer Mravlag, Erik Haehl, Simon K. B. Spohn, Constantinos Zamboglou, Peter E. Huber, Jürgen Debus, Anca-Ligia Grosu, Tanja Sprave, Nils H. Nicolay

**Affiliations:** 1 Department of Radiation Oncology, University of Freiburg—Medical Center, Robert-Koch-Str. 3, 79106 Freiburg, Germany; 2 German Cancer Consortium (DKTK) Partner Site Freiburg, German Cancer Research Center (DKFZ), Neuenheimer Feld 280, 69120 Heidelberg, Germany; 3 Department of Molecular Radiation Oncology, German Cancer Research Center (DKFZ), Neuenheimer Feld 280, 69120 Heidelberg, Germany; 4 Department of Radiation Oncology, University Hospital Heidelberg, Im Neuenheimer Feld 400, 69120 Heidelberg, Germany

**Keywords:** Low-dose radiotherapy, Elderly patients, Osteoarthritis, Joint disorder, Benign diseases

## Abstract

**Purpose:**

Painful osteoarthritis is common in elderly patients, and low-dose radiotherapy has been demonstrated to provide effective symptomatic treatment. We examined the analgesic effects of low-dose radiotherapy for osteoarthritis in the elderly aiming to reveal potential differences in the response rates relating to increasing age.

**Methods:**

A retrospective analysis was performed at two university hospitals including elderly patients (≥ 65 years) undergoing radiotherapy for osteoarthritis between 2008 and 2020. Pain intensity and response were quantified using the numerical rating scale (NRS) and the Pannewitz score. Age groups were defined for young old (65–74 years), older old (75–84 years), and oldest old patients (≥ 85 years).

**Results:**

In all, 970 patients with 1185 treated sites and a median age of 76 years were analyzed. Mean NRS was 66 at baseline (t0), 53 after radiotherapy (t1), and 44 at first follow-up (t2) (*p* < 0.001 for t0–t1, t1–t2, and t0–t2). At t1, 1.5% exhibited a Pannewitz score of 0 (no pain), 58.5% of 1–2 (less pain), 36.1% of 3 (equal pain), and 3.9% of 4 (worse pain), while at t2, pain response shifted towards 6.9% (0), 58.6% (1–2), 28.1% (3), and 6.3% (4). Pain response did not differ between age groups at t1 (*p* = 0.172) or t2 (*p* = 0.684). In addition, pain response after re-irradiation (*n* = 384 sites) was 61.0% and was comparable between age groups (*p* = 0.535).

**Conclusion:**

Low-dose radiotherapy results in pain reduction in about two-thirds of treated sites with no difference relating to increasing age, showing that radiotherapy is an effective analgesic treatment for osteoarthritis even at advanced ages.

## Introduction

Osteoarthritis is a common disease especially in the elderly that is becoming more frequent in developed countries due to an increasing life expectancy [1]. Osteoarthritis is the most frequent joint disease in adults globally, and about one in three adults exhibits radiological signs of osteoarthritis [2, 3]. Considering the reduction of health-related quality-of-life in affected patients, the considerable socioeconomic costs due to multiple therapeutic procedures, and the secondary complications, e.g., opioid abuse, psychological disorders, physical inactivity and thereby increased risk for obesity and cardiovascular diseases, osteoarthritis has a huge impact on the health systems [4–7]. There are several therapeutic options with varying treatment intensity including physical therapy, nonsteroidal anti-inflammatory drugs (NSAIDs), intraarticular corticosteroid or hyaluronic acid injection and surgical procedures such as joint replacements or arthrodesis [8].

Due to the anti-inflammatory effects of small doses of ionizing radiation, low-dose radiotherapy is commonly used in patients with painful osteoarthritis, especially in central and Eastern Europe [9]. The advantages of low-dose radiotherapy include the noninvasive nature, the cost-efficacy, and the near absence of toxicities. As the risk for radiation-induced tumors using low radiation doses is very low and further reduced in elderly patients, low-dose radiotherapy for osteoarthritis is an attractive treatment in this cohort [10, 11]. The anti-inflammatory effect of low-dose radiotherapy has been shown to be mediated by several effects such as the modulation of expression of endothelial cells’ adhesion molecules, cytokine release by leukocytes and nitric oxide production by macrophages [12–16]. Furthermore, low-dose radiotherapy was found to positively influence bone metabolism by increasing osteoblast-induced mineralization and decreasing RANK‑L levels [17].

A plethora of studies have reported analgesic effects of low-dose radiotherapy for osteoarthritis, but these studies mostly did not focus on the important subgroups of elderly patients [18–25]. Nevertheless, there are several differences between younger and elderly osteoarthritis patients [26–28]: Osteoarthritis is based on a continuous degenerative process that is therefore more pronounced in advanced ages. In turn, the proportion of patients with posttraumatic secondary osteoarthritis is lower in elderly patients. Elderly patients exhibit a different pain perception than younger patients, and the duration of osteoarthritis-related pain generally is longer in elderly patients [26–28]. A previous prospective trial reported beneficial effects of low-dose radiotherapy in elderly patients with painful skeletal disorders; however, patients with osteoarthritis of the hands or the shoulder were not included in the study [29].

We therefore aimed to analyze the effects of linear accelerator-based low-dose radiotherapy in a large cohort of elderly osteoarthritis patients treated at two university hospitals.

## Material and methods

### Treatment

The Independent Ethics Committees of University of Freiburg (reference no. 150/20) and University of Heidelberg (reference no. S‑040/2018) approved this study. Patients who received low-dose radiotherapy for osteoarthritis between 2008 and 2020 and were ≥ 65 years at the time of radiotherapy were included. Low-dose radiotherapy was applied for painful osteoarthritis of the large joints, i.e., the knees, hips and shoulders, as well as the small joints, i.e., the wrist, fingers, thumbs, ankle, and feet, following the guidelines of the German Society of Radiation Oncology [30]. Radiotherapy was regularly performed in 6 fractions with single doses of 0.5 or 1 Gy that were given twice or thrice weekly. All patients received low-dose photon radiotherapy using a linear accelerator either after computed tomography (CT)-based 3‑dimensional treatment planning or after treatment simulation using 2‑dimensional X‑ray imaging. Initial pain intensity prior to treatment (t0) as quantified by the numerical rating scale (NRS) and preceding therapeutic procedures such as NSAID intake or intraarticular corticosteroid injections were extracted from the patient records. Initial pain response immediately after radiotherapy (t1) was accessible for all patients using the Pannewitz score ranging from 0 (complete pain relief) to 4 (worsening of pain). As the retrospective data did not always allow to differentiate between the Pannewitz score 1 (major pain relief) and 2 (minor pain relief), we summarized both score points. Patients were invited for a follow-up consultation at about 8 weeks after completion of radiotherapy (t2), and in case of inadequate response or recurrent pain, a second radiotherapy course was discussed with the patients.

### Statistical analysis

Wilcoxon signed-rank tests were used to compare NRS values between the time points. Mann–Whitney U (2 groups) or Kruskal–Wallis tests (≥ 3 groups) were performed to compare the pain response measured by the Pannewitz score. A χ^2^ test was performed to examine whether the re-irradiation rate was different between the age groups. Statistical significance was assumed for *p* *<* 0.05. IBM SPSS Statistics software version 25 (IBM, Armonk, NY, USA) and GraphPad Prism software version 8 (GraphPad Software, San Diego, CA, USA) were used for statistical analyses.

## Results

### Patient and treatment characteristics

A total of 970 patients with 1185 treated lesions were analyzed. As some patients were treated for separate joints at different time points, patient characteristics are related to the total number of sites (Table 1). Median age of our elderly cohort was 76 years (range 65–98 years), and almost three quarters of patients were female (*n* = 858, 72.4%). Median body mass index (BMI) was assessable for 270 patients and amounted to 27.7 kg/m^2^ (range 16.0–52.1 kg/m^2^). Most common treatment sites included knees (*n* = 419, 35.4%), hands (*n* = 363, 30.6%), and feet (*n* = 219, 18.5%). The majority of patients were treated with NSAIDs prior to radiotherapy (*n* = 733, 61.9%), and a considerable percentage of patients had also received prior intraarticular corticosteroid injections (*n* = 221, 18.6%). The most common treatment scheme was 6 × 1 Gy (*n* = 916, 77.3%), while 6 × 0.5 Gy was less frequently used (*n* = 257, 21.7%).

**Table 1:** Patient and treatment characteristics of the study cohort (*n* = 1185 joints)

		Median (range)	
Age at radiotherapy (years)		76 (65–98)	
Body mass index (kg/m^2^), *n* = 270		27.7 (16.0–52.1)	
		*n*	%
Age groups	65–74 years	544	45.9
	75–84 years	507	42.8
	≥ 85 years	134	11.3
Gender	Male	327	27.6
	Female	858	72.4
Location	Hand	363	30.6
	Shoulder	147	12.4
	Hip	33	2.8
	Knee	419	35.4
	Foot	219	18.5
	Others	4	0.3
Previous therapeutic measures	NSAIDs	733	61.9
	Intraarticular corticosteroid injection	221	18.6
Radiotherapy fractionation	6 × 0.5 Gy	257	21.7
	6 × 1 Gy	916	77.3
	Others	12	1.0

### Pain response

**Fig. 1:**
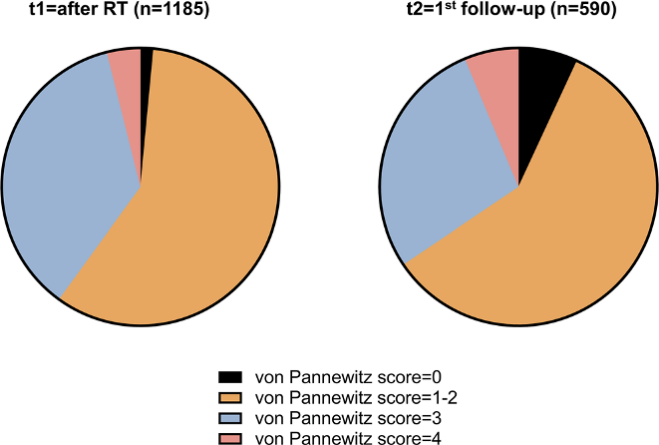
Two-thirds of elderly patients exhibit pain improvements after low-dose radiotherapy (RT) for osteoarthritis. Pie chart showing the distribution of the Pannewitz score at t1 (after the last radiation fraction) and at t2 (at the first follow-up)

Directly upon completion of low-dose radiotherapy, 18 patients (1.5%) exhibited a complete pain relief (Pannewitz score = 0), 693 (58.5%) reported a partial response (Pannewitz score = 1–2), 428 (36.1%) unaltered pain (Pannewitz score = 3), and 46 (3.9%) increases in pain, therefore resulting in a response rate (Pannewitz score 0–2) of 60.0% at t1 (Fig. 1). In all, 590 patients (49.8%) presented for the first follow-up consultation or filled out paper-based pain questionnaires (t2): 387 patients (65.6%) had a pain response, whereas 203 patients (34.4%) reported no pain response. Of these 203 patients, 166 patients (28.1%) exhibited stable pain and 37 (6.3%) showed increases in pain intensity. Mean NRS was 66.0 (±11.1) prior to radiotherapy, 53.4 (±18.0) at t1 and 44.5 (±23.7) at t2 (*p* < 0.001 for t0–t1, t1–t2 and t0–t2, Wilcoxon signed-rank test). By only analyzing patients with information at t0, t1, and t2, the mean NRS difference amounted to −12.3 (±15.4) between t0 and t1 and −21.0 (±23.9) between t0 and t2.

**Fig. 2:**
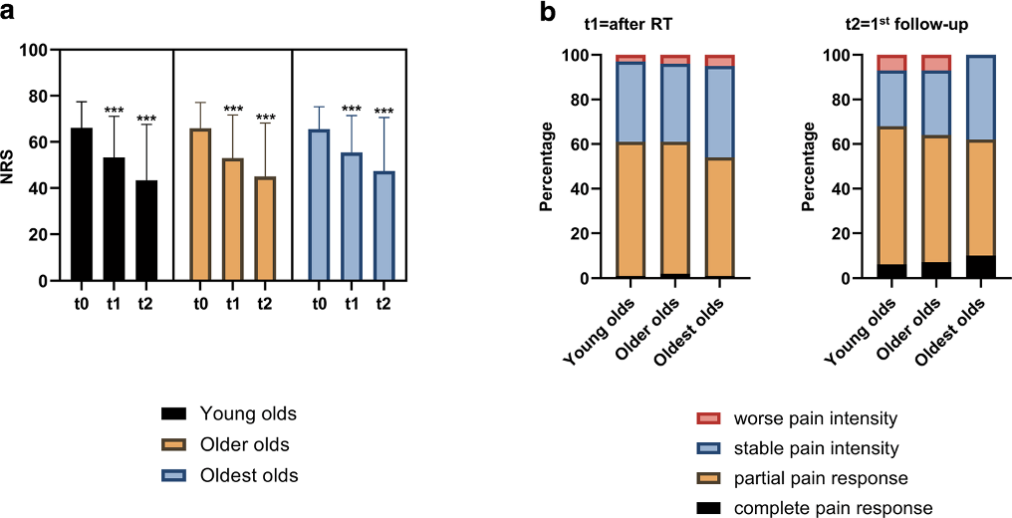
Pain response is not dependent on patient’s age in elderly patients with painful osteoarthritis. **a** Mean numerical rating scale (NRS) values with the according standard deviation are shown for the three age groups (young olds = 65–74 years, older olds = 75–84 years, oldest olds = 85 years and older) at the different time points. T0 = NRS prior to radiotherapy (RT), t1 = NRS at the last radiation fraction, t2 = NRS at the first follow-up. Wilcoxon signed-rank tests were used to compare t1 with t0 and t2 with t0. ***p < 0.001. **b** Pannewitz scores at t1 and t2 in dependence of patient’s age. Kruskal–Wallis tests did not reveal significant differences regarding the Pannewitz score between the different age groups

Following a common subdivision approach for the elderly population, we divided our cohort into young olds (65–74 years), older olds (75–84 years), and oldest olds (≥ 85 years) and compared pain response rates among the three age groups (Fig. 2). Initial mean NRS values were comparable and ranged at 66.1, 65.9, and 65.5 for young olds, older olds, and oldest olds, respectively (*p* = 0.759, Kruskal–Wallis test). Both at t1 and t2, there were no significant differences regarding patients’ NRS between the different age groups (t1: *p* = 0.467, t2: *p* = 0.477). Similarly, the Pannewitz score was found to be similar between the different age groups of elderly osteoarthritis patients: 6 (1.1%), 325 (59.7%), 195 (35.8%), and 18 (3.3%) young old patients exhibited Pannewitz scores of 0, 1–2, 3, and 4, respectively at t1. This was comparable to the distribution in the older olds (2.2% with Pannewitz = 0, 58.8% with Pannewitz = 1–2, 34.9% with Pannewitz = 3, and 4.1% with Pannewitz = 4) and oldest olds (0.7% with Pannewitz = 0, 52.2% with Pannewitz = 1–2, 41.8% with Pannewitz = 3, and 5.2% with Pannewitz = 4) (*p* = 0.172, Kruskal–Wallis test). At t2, there also were no significant differences regarding Pannewitz scores between the three age groups (*p* = 0.684). The overall pain response rates (Pannewitz score = 0–2) at t2 were 68.0%, 64.0%, 61.5% for the young olds, older olds, and oldest olds, respectively.

**Fig. 3:**
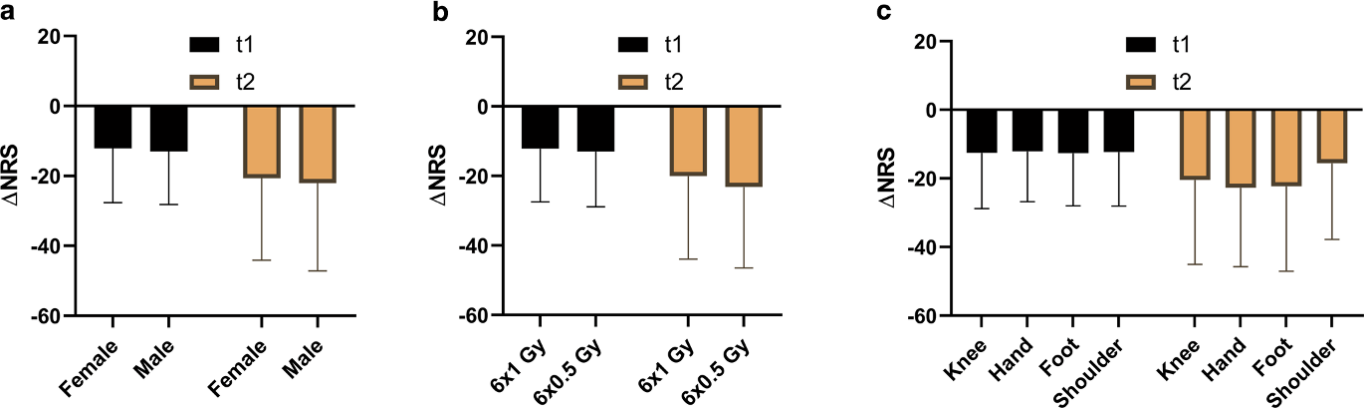
Low-dose radiotherapy reduces pain intensity irrespectively of gender, fractionation and joint location in the elderly. Pain response ΔNRS at t1 = immediately upon radiotherapy completion and t2 = first follow-up consultation compared to baseline NRS value in dependence of gender (**a**), radiotherapy fractionation (**b**) or osteoarthritis location (**c**). *NRS* numerical rating scale

We furthermore analyzed the role of patient gender, radiotherapy fractionation, and osteoarthritis location on the pain response (Fig. 3). Prior to radiotherapy, female patients had higher NRS values than male patients (66.9 versus 63.6, *p* < 0.001, Mann–Whitney U test). However, there were no differences in the mean NRS decline between male and female patients at t1 (*p* = 0.271) or t2 (*p* = 0.610). As shown in previous prospective trials for other benign diseases, there was no difference in the analgesic efficacy between single doses of 1 Gy and 0.5 Gy (t1: *p* = 0.313, t2: *p* = 0.178, Mann–Whitney U test) [31–33]. The different osteoarthritis sites in our study showed similar NRS dynamics both at t1 and at t2 with no differences between the groups (t1: *p* = 0.970, t2: *p* = 0.192, Kruskal–Wallis test). Neither at t1 (*p* = 0.336, Mann–Whitney U test) nor at t2 (*p* = 0.380), patients with previous NSAID intake had a different pain response as indicated in the Pannewitz score distribution. Similarly, previous intra-articular corticosteroid administration had no effect on patients’ pain response (t1: *p* = 0.361, t2: *p* = 0.273). Patients with obesity (BMI > 25 kg/m^2^) exhibited similar NRS declines both at t1 (−10.9 versus −9.4, *p* = 0.412) and at t2 (−20.6 versus −21.0, *p* = 0.690) compared to patients of normal weight. Likewise, pain responses as measured with the Pannewitz score were not different between overweight and normal-weight individuals (t1: *p* = 0.970, t2: *p* = 0.617).

### Second radiotherapy course

**Fig. 4:**
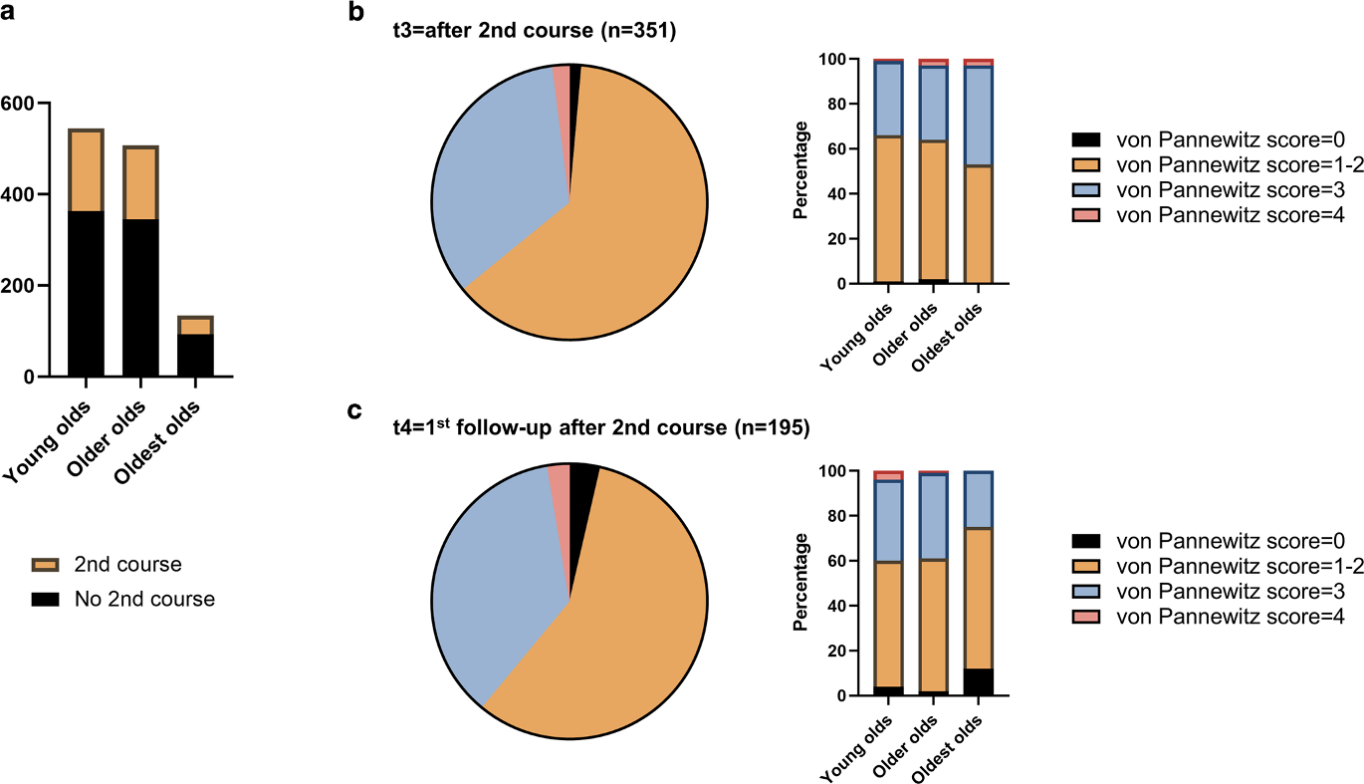
Second courses of low-dose radiotherapy result in still favorable pain response rates in the elderly.** a** Frequency of re-irradiation in the young olds (65–74 years), older olds (75–84 years), and oldest olds (≥ 85 years). **b** Pain response determined by the Pannewitz score at t3 = the last fraction of the second course. **c** Pannewitz score distribution at t4 = first follow-up after re-irradiation

We also investigated whether there were differences in the frequency of second radiotherapy courses depending on patient age. A total of 384 s radiotherapy courses (32.4%) were applied in our cohort. Re-irradiation rates did not differ between the different age groups and amounted to 33.3% in the young olds, 32.0% in the older olds, and 30.6% in the oldest olds (*p* = 0.805, χ^2^ test; Fig. 4). In a total of 351 treated sites, pain response could be assessed at the end of the second radiotherapy course (t3). At t3, complete pain relief (Pannewitz score = 0) was found in 5 cases (1.4%), partial response (Pannewitz score = 1–2) in 220 cases (62.7%), stable pain (Pannewitz score = 3) in 119 cases (33.9%), and worse pain (Pannewitz score = 4) in 7 cases (2.0%). Again, there were no differences in the Pannewitz score distribution between the three age groups (*p* = 0.256, Kruskal–Wallis test). At the first follow-up after the second course (t4, *n* = 195), Pannewitz score distribution was similar to t3: 3.6% of patients had a Pannewitz score = 0, 57.4% a Pannewitz score = 1–2, 36.4% a Pannewitz score = 3, and 2.6% a Pannewitz score = 4, leading to a pain response rate of 61.0% after re-irradiation that was comparable between the age groups (*p* = 0.535, Kruskal–Wallis test).

## Discussion

We demonstrated in a large multicenter cohort including 970 elderly patients with 1185 treated sites that low-dose radiotherapy is an analgesic treatment for painful osteoarthritis irrespectively of patient age. Our study is, to the best of our knowledge, the largest study of elderly osteoarthritis patients receiving low-dose radiotherapy. We did not observe differences regarding the NRS or the Pannewitz score dynamics between young old, older old, and oldest old patients, showing that low-dose radiotherapy does not lose efficacy in patients with very advanced age.

Considering that very old patients have a higher risk for toxicities from long-term NSAID intake (e.g., gastrointestinal bleeding [34]), low-dose radiotherapy can be considered as appropriate alternative for elderly osteoarthritis patients. Furthermore, very old osteoarthritis patients bear a considerably reduced carcinogenic risk after low-dose radiotherapy considering the proposed latency time of solid cancer induction. The overall pain response rate at the first follow-up appointment in our study was 65.6% which is in the range or slightly lower than reported in previously published studies in which a case-related average of 77.7% pain response was reported [24]. Furthermore, the re-irradiation rate in our cohort amounted to 32.4% which is comparable to the 30% rate reported in a German patterns-of-care study for painful knee osteoarthritis [9]. Compared to other studies, the average age of 76 years in our cohort was considerably higher (e.g., Hautmann et al.: 65 years [24], Micke et al.: 63 years [23], Keilholz et al.: 64 years [35], Minten et al.: 65 years [36], Kaltenborn et al.: 62 years [37]). Importantly, we could not find subgroups of elderly patients that benefitted less from low-dose radiotherapy, and neither gender, fractionation, joint location, previous NSAID administration, intra-articular corticosteroid injections, nor BMI influenced pain response rates.

Although a placebo effect cannot be ruled out, it should be considered that low-dose radiotherapy is commonly applied for patients after multiple previous therapy attempts, therefore considering a rather intensively pretreated patient cohort with a long disease history. Increasing pain response rates between t1 and t2, as seen in our study, would also rather be untypical for a pure placebo effect. It may further be hypothesized that elderly patients with favorable pain response after low-dose radiotherapy would rather omit the follow-up consultation at t2, thereby biasing the results towards lower pain response rates. In turn, patients that exhibit equal or even more intense pain after radiotherapy could be maybe more likely to keep the appointment at the follow-up consultation to receive a second course.

Although low-dose radiotherapy is a widely used treatment modality for osteoarthritis in central and Eastern Europe and numerous retrospective analyses have demonstrated the pain-relieving effects of this therapy, two randomized, double-blind, sham-controlled studies could not confirm superior analgesic effects of low-dose radiotherapy for knee and hand osteoarthritis compared with sham treatment [36, 38]. However, low patient numbers, an inadequate power to detect moderate benefits, short follow-up times, imbalances in the treatment groups, and the inclusion of patients with severe pain and long histories of chronic pain suggesting already advanced joint degeneration are considered as limitations of these studies [39]. Older randomized trials had similarly short follow-up times and included indications such as intercostal neuralgia and spondylitis which no longer constitute indications for low-dose radiotherapy [40, 41]. Therefore, there is an urgent need for adequately designed high-quality randomized controlled trials with sufficient patient numbers and follow-up times as well as an appropriate patient selection in order to improve the evidence for low-dose radiotherapy of osteoarthritis [42].

Although providing real-world data of low-dose radiotherapy for painful osteoarthritis in a large cohort of elderly patients, our analysis has limitations: For instance, about half of patients failed to present for the first follow-up appointment; therefore, information on patient’s pain intensity at t2 of these patients are missing. Second, we had insufficient data about patients’ long-term pain response, as patients were not routinely re-assessed over the following years. However, previous analyses rather have shown that pain response is augmented with increasing duration and that the full analgesic effect of low-dose radiotherapy is most pronounced at later time points [43].

## Conclusion

To the best of our knowledge, this is the largest study of elderly osteoarthritis patients treated by low-dose radiotherapy. Our data demonstrate that low-dose radiotherapy is an effective treatment in elderly patients with osteoarthritis, resulting in pain improvement in two of three cases. As we could not detect reduced analgesic efficacy in the older/oldest old patients, our data suggest that there is no upper age limit for radiotherapy. As the risk for tumor induction is decreasing with advancing age, low-dose radiotherapy may even have a more prominent role in this population.
